# The relationship between the intake of branched-chain and aromatic amino acids and individuals' sleep quality based on body mass index, gender, and age

**DOI:** 10.1186/s41043-023-00383-5

**Published:** 2023-05-26

**Authors:** Sahar Noori, Maryam Nadery, Rasool Ghaffarian-Ensaf, Alireza Khadem, Khadijeh Mirzaei, Seyyed Ali Keshavarz, Ariyo Movahedi

**Affiliations:** 1 Department of Nutrition, Science and Research Branch, Islamic Azad University, Tehran, Iran; 2 Ph.D Student, Florida International University, Miami, USA; 3 Department of Community Nutrition, School of Nutritional Sciences and Dietetics, Tehran University of Medical Sciences (TUMS), Tehran, Iran; 4 Department of Clinical Nutrition, School of Nutritional Sciences and Dietetics, Tehran University of Medical Sciences (TUMS), Tehran, Iran

**Keywords:** Branched-chain amino acids, Aromatic amino acids, Sleep quality

## Abstract

Sleep disorder is a relatively common problem that causes chronic conditions such as obesity, diabetes, and cardiovascular diseases. It is believed that diet regulates sleep. So, investigating the relationship between branched-chain amino acids (BCAAs) and aromatic amino acids intake with sleep quality based on age, gender and Body Mass Index (BMI) is important. A total of 172 males and females aged 18–65 participated in this study. The questionnaires were given online to them, included demographic information, food frequency questionnaire (FFQ), International Physical Activity Questionnaire, and Pittsburgh Sleep Quality Index. The Chalder fatigue scale (CFQ) was also used to measure the extent and severity of fatigue. The intake of amino acids was investigated by FFQ. The relationship between amino acids intake and sleep quality was investigated using Pearson's test. The results showed that intake of energy, macronutrients, and some micronutrients had a significant relationship with the quality of sleep of men compared to that of women (*P* < 0.05). No difference in sleep duration was observed between the two genders. There was a significant, positive association between sleep duration and the intake of BCAA (CC = 0.205, *P* = 0.031) and aromatic amino acids (CC = 0.22, *P* = 0.02) in the participants with normal BMI. Significant differences were seen in the intake of BCAA according to BMI which these differences were between lean and obese people, lean and overweight people, obese and normal, and overweight people. It demonstrated that in individuals with normal BMI, amino acids, protein, and carbohydrate intake may affect sleep duration and with modification of these factors sleep quality may get better. More study is needed to confirm these findings.

## Introduction

Sleep disorder is a relatively common problem that disrupts the body's natural circadian rhythm, affecting psychological and physical health [1]. Sleep is a natural state of rest and a primary physiological process regulating the body's health status [2, 3]. In addition, it is a crucial index to examine individuals’ health status [4]. The body maintains a circadian biological rhythm called the circadian rhythm. It regulates the sleep–wake cycle and repeats roughly every 24 h [5]. It is controlled by the genetic components of the biological clock (clock genes) and peripheral factors like nutritional and environmental ones [6]. Sleep problems have constituted a global epidemic during the last 40 years. The average sleep duration has declined by 2 h [7].

Moreover, global sleep dissatisfaction increases with advancing age, which is higher among women than men [8]. Approximately 30% of adults suffer from one or more insomnia symptoms [9]. Several factors, including environmental pollution, socio-economic factors [10], employment status, physical activity, psychiatric problems and eating habits, affect sleep [11]. The negative effect of insufficient sleep on the brain's structure, activity, and physiology has been reported in many studies [12–14]. Insufficient sleep, quantitatively and qualitatively, can increase the risk of chronic diseases like obesity, metabolic disorders, diabetes, and cardiovascular diseases [15]. Many studies have shown the relationship between poor sleep quality with increased appetite, uncontrollable eating [16], and unhealthy eating habits [17], and diet balance [18, 19]. Thus, poor sleep quality is associated with poor daytime performance and is a risk factor for physical or mental disorders [20]. Protein is one of the controversial nutrients which affects sleep [21]. Tryptophan is the sole precursor of serotonin, which can improve sleep. Moreover, the researchers have found that high evening dietary protein intake leads to increased alertness because of the stimulating effect of specific amino acids on adrenaline and noradrenaline [22, 23] and competition in the transport of tryptophan from the blood–brain barrier with Large neutral amino acids (LNAAs) [24].

Recent investigations, especially on the population suffering from sleep disorders, have introduced nutrition as one of the main options for improving sleep [25]. Nutrients and physical activity play a key role in regulating the body's internal clock and determining sleep quality [26, 27]. Hashimoto and colleagues (2020) conducted a cross-sectional study on the relationship between dietary quality and energy intake and women's sleep quality. They reported that protein intake was significantly lower in the low-sleep efficiency (SE) group than in the high-SE group [20]. Saidi and colleagues (2019) studied the effect of proteins with different tryptophan/large neutral amino acid ratios on sleep in adolescents. They concluded that a non-pharmacological approach based on nutritional quality (protein with tryptophan /Large neutral amino acids ratio (Trp/LNAAs)) could significantly improve sleep duration/ quality [21].

Considering what was mentioned above, the present study investigates the relationship between the intake of branched-chain amino acids (BCAAs) and aromatic amino acids with sleep quality based on body mass index (BMI), gender, and age.

## Materials and methods

### Participants

Participants aged 18–65 were recruited and screened based on the following inclusion/exclusion criteria. The inclusion criteria: (1) age between 18 to 65 years old, (2) did not have physical or mental disabilities, (3) not having an amputation or recent surgery. Exclusion criteria: (1) not taking sleeping pills, (2) not being pregnant or lactating. The research population included people living in Tehran.

### Study design

In the present cross-sectional study, sampling and data collection were performed in 2020–2021. Random sampling was used. The sample size was calculated using GPower software version 3.1.9.7 (Faul, 2014) with the settings of Linear Bivariate Regression studies and Correlation: Bivariate model studies where the highest number was equal to 138 individuals. With a 20% probability of withdrawal, a total of 166 people was considered a sample. Questionnaires were sent to individuals online. This study was conducted according to the guidelines laid down in the Declaration of Helsinki, and all procedures involving human subjects were approved by the ethical Iran National Committee for Ethics in Biomedical Research with the following identification IR.IAU.SRB.REC.1400.106. Written informed consent was obtained from all subjects/patients.

### Assessment of anthropometric measurement

The questionnaires were given online to the participants, and the required data were collected. Body weight and height were asked of individuals through an online demographic questionnaire.

### Assessment of dietary intake, sleep quality, fatigue, and physical activity

Fatigue was measured using Chalder Fatigue Scale (CFS) (alpha-Cronbach coefficient: 0.82) to measure the extent and severity of physical and mental fatigue. The validity and reliability of the CFS questionnaire have been confirmed by Nasri and colleagues [28]. Pittsburgh Sleep Quality Index (PSQI) (alpha-Cronbach coefficient: 0.77) and 147-item semiquantitative food frequency questionnaire (FFQ) (alpha-Cronbach coefficient: 0.93) were used to assess sleep quality and disturbances and calculate nutrient and calorie intake, respectively. FFQ questionnaire consists of a list of 147 food items with a standard serving size for each item, designed based on the Willett- format and used in the previous studies by Ismailzadeh, Azadbakht, Mirmiran and others to determine food patterns. Its validity and reliability have been evaluated over and over [29]. Food intakes were reported and recorded yearly, monthly, weekly, or daily and then converted to g per day using household measurements. Using the residual method, the obtained amounts were adjusted for energy intake [30]. To evaluate participants’ dietary intakes, trained interviewers used the 147 Quantitative Food Frequency Questionnaire. Using NUTRITIONIST 4 (First Data Bank, San Bruno, CA) software, dietary intakes were analyzed. PSQI scale has several components, which include subjective sleep quality, sleep latency, sleep duration, sleep efficiency, sleep disturbance, use of sleep medication, and daytime dysfunction. The sum of the scores of 7 subscales will be between 0 and 21. On each scale, a person's score will be between 0 and 3, which are interpreted as follows: no sleep problem: score 0, moderate sleep problem: score 1, serious sleep problem: score 2, very serious sleep problem: score 3. Obtaining a higher total score out of 5 in the whole questionnaire means poor sleep quality. PSQI questionnaire has been used several times in Iran [31, 32]. Participants' online responses to the short form of the International Physical Activity Questionnaire (IPAQ) (alpha-Cronbach coefficient: 0.78) were collected and reported as metabolic equivalent minute/week (MET-min/wk) [33]. This questionnaire calculates the physical activity of all participants during the past 7 days. IPAQ included five sections of questions in physical activity: (1) work-related physical activity, (2) housework, (3) transportation, (4) physical activity during leisure -time and sports, (5) and the time spent sitting. Participants responded according to the intensity (moderate or severe) and the length of time they were engaged in these activities during the last seven days. Then, according to guidelines, the values were multiplied by their MET quantities, and the acquired numbers were summed together to calculate MET/min/week values [34]. IPAQ questionnaire has been used in various studies in Iran, and its validity and reliability have been confirmed [35].

### Statistical analysis

Data were analyzed using Statistical Package for Social Sciences (version: 26; SPSS Inc, Chicago, IL, USA), and a *P* value less than 0.05 (*P* < 0.05) was considered statistically significant. The Shapiro–Wilk test was used to evaluate the normality of the quantitative variables.

Quantitative variables including age, height, weight, and BMI were described as mean ± standard deviation (SD), using the independent t test and for categorical variables frequency (%) was used. To compare the intake of BCAA and aromatic amino acids among the participants, and to compare sleep quality, sleep duration and physical activity based on gender, age and BMI, ANOVA test and Duncan's Multiple Range test were used. The dietary data obtained in the study were first entered into the Nutritionist software (N4), and then, the results were analyzed using the statistical software SPSS v26. Pearson's correlation was used to determine the relationship between the intake of BC and aromatic amino acids with the variables based on gender, age, and BMI.

## Results

### Study population characteristics

**Fig. 1:**
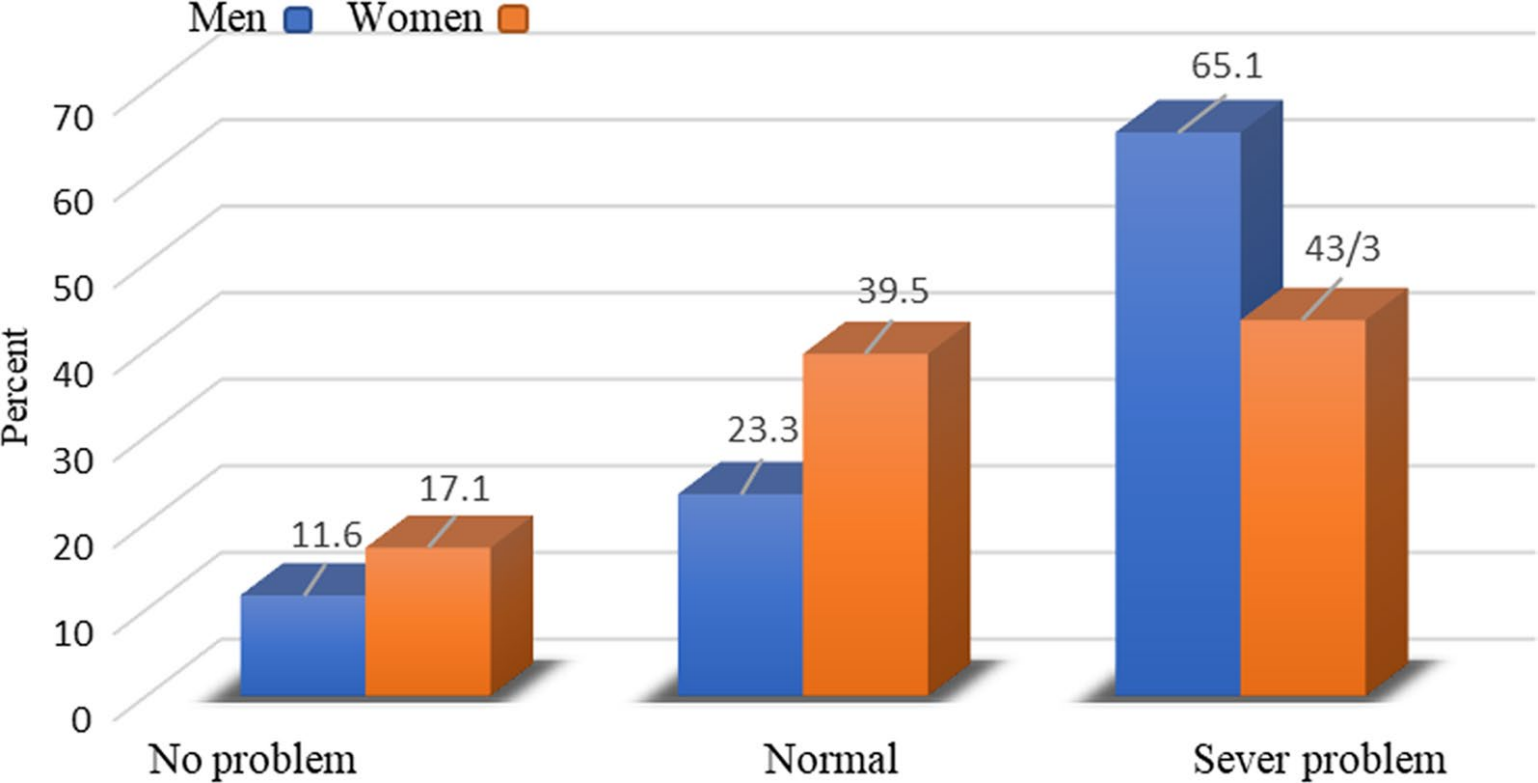
Comparison of the frequency of sleep quality scores of individuals based on gender

**Fig. 2:**
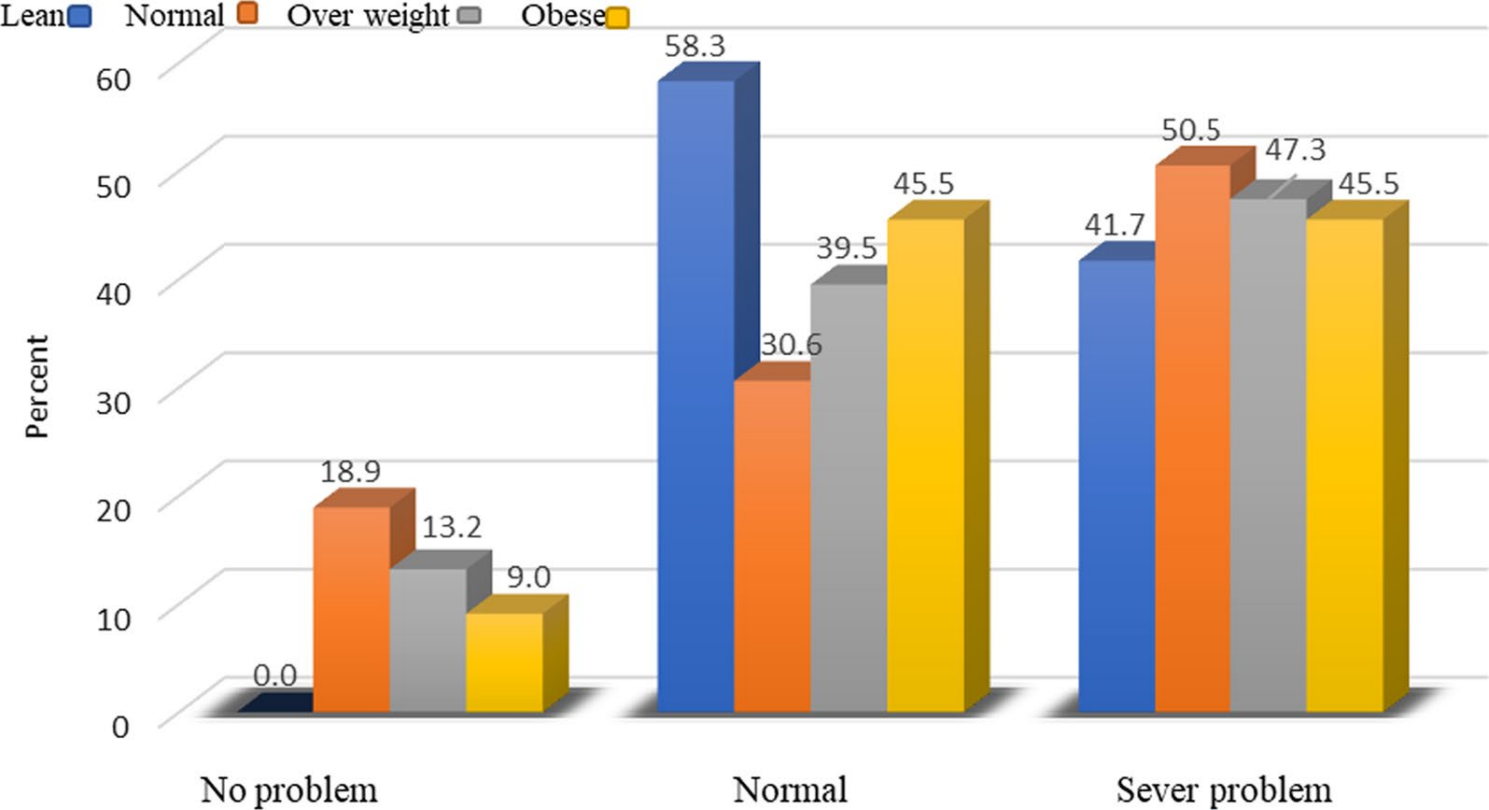
Comparison of the frequency of sleep quality score of individuals based on BMI

The mean (SD) of the age of the total subjects was 29.81 (9.82), height 166.25 (8.68), weight 65.04 (14.92), BMI 23.32 (3.93), sleep quality score 11.064 (10.47), sleep duration 6.95 (1.44) and physical activity 1927.92 (989.1). 79.1% of subjects had high education (bachelor's degree to PhD) and 20.4% had low education status (diploma and under diploma). The results obtained from the present study showed that 65.1% of men and 43.4% of women suffered from severe sleep problems. In fact, men suffered from more serious sleep problems compared to women, based on their PSQI score mentioned in Fig. 1. Also, in Fig. 2, 50.5% of people with a normal BMI in the first place and 47.3% of overweight people experienced such problems.

No significant difference was observed between severe sleep problems in people under 30 years old and people over the same age. 49.2% of people under 30 and 48.1% over 30 years old suffered from severe sleep problems, respectively. Regarding sleep duration based on gender, 70% of men and 72.8% of women had adequate sleep, but 14% of men and 13.2% of women had sleep deprivation. The frequency percentage of sleep duration based on BMI showed that 77.5% of people with normal BMI had adequate sleep, and 9.9% experienced poor sleep. Moreover, 23.7% of overweight people had sleep deprivation, and 60.5% had adequate sleep. 72% of people under 30 years old and 1.74% of people over 30 had adequate sleep.

### Sleep quality, fatigue, and physical activity of the participants based on their gender

The scores from Chalder mental and physical fatigue scale presented in Table 1 showed a significant difference between men and women. In other words, men's sleep quality was better than women's (*P* = 0.007), but the sleep duration showed no difference between genders. However, physical (*P* = 0.001) and mental fatigue (*P* = 0.017) were more in women compared to men.

**Table 1:** The sleep quality, fatigue, and physical activity of the participants based on their gender

Variables	Male (*N* = 42)	Female (*N* = 118)	*P* value
Sleep duration (h)	6.86 ± 1.52	6.98 ± 1.41	0.626
The score of sleep quality	14.74 ± 11.62	9.84 ± 9.81	**0.007**
Chalder physical fatigue scale	11.02 ± 5.82	15.43 ± 5.60	**0.001**
Chalder mental fatigue scale	17.63 ± 3.37	9.08 ± 3.44	**0.017**
Physical activity (MET-min/wk)	1689.96 ± 973.18	2007.25 ± 985.32	0.068

Quantitative variables were reported as mean ± SD

*P* values resulted from the analysis of independent t test for continuous variables

*P* value < 0.05 was considered significant. Bold values are significant

### Sleep quality, fatigue, and physical activity of the participants based on their age

In Table 2, no significant difference was observed between the sleep quality and fatigue of people based on age; however, the amount of physical activity in people under 30 years old was significantly higher than in people over the same age (*P* = 0.005).

**Table 2:** Comparing the sleep quality, sleep duration, and physical activity among the participants based on age

Variables	Over 30 years old (*N* = 54)	Under 30 years old (*N* = 118)	*P* value
Sleep duration (h)	6.89 ± 1.37	6.98 ± 1.47	0.691
The score of sleep quality	9.13 ± 7.43	11.95 ± 11.52	0.101
Chalder physical fatigue scale	13.66 ± 6.23	14.64 ± 5.84	0.324
Chalder mental fatigue scale	8.28 ± 3.55	8.92 ± 3.44	0.265
Physical activity (MET-min/wk)	1619.26 ± 702.65	2069.18 ± 1068.70	**0.005**

Quantitative variables were reported as mean ± SD

*P* values resulted from the analysis of independent t test for continuous variables

*P* value < 0.05 was considered significant﻿. Bold values are significant

There was no significant difference in the intake of both amino acids groups, the sleep duration/quality.

### Comparing the sleep quality, sleep duration, physical activity, and intake of BCAAs and aromatic amino acids among the participants based on BMI

According to Table 3, a significant difference in the intake of BCAAs between lean and obese participants and overweight participants was observed on the one hand and obese, normal, and overweight participants. There was a significant difference in the intake of aromatic amino acids between lean and overweight participants, lean and obese, and normal and obese participants. Also, a significant difference in physical activity was observed. In this way, the average physical activity between obese and overweight participants, obese and normal participants, obese and lean participants, lean and overweight participants and lean and normal participants was significantly different. Sleep quality and sleep duration were not significant based on BMI.

**Table 3:** Comparing the sleep quality, sleep duration, physical activity, intake of branched-chain amino acids, and aromatic amino acids among the participants based on BMI

Variables	BMI			
	Lean (*N* = 12) (BMI < 18.5)	Normal (*N* = 111) (BMI = 18.5–24.9)	Overweight (*N* = 38) (BMI = 25–29.9)	Obese (*N* = 11) (BMI ≥ 30)
Branched-chain amino acids (mg/d)	9010.10 ± 3575.52^a^	12,480.53 ± 6729.88^ab^	14,220.13 ± 5544.74^bc^	18,865.13 ± 12,427.73^d^
Aromatic amino acids (mg/d)	6020.47 ± 2229.68^a^	7965.71 ± 3578.58^ab^	9292.06 ± 3603.08 ^bc^	11,494.56 ± 6253.28^cd^
Sleep quality	11.08 ± 10.38	11.19 ± 10.47	11.18 ± 11.07	9.36 ± 9.66
Sleep duration (h)	7.67 ± 1.15	7.01 ± 1.32	6.66 ± 1.74	6.64 ± 1.50
Physical activity (MET-min/wk)	3019.62 ± 1474.13^a^	2011.71 ± 888.61^b^	1657.21 ± 823.18^cd^	826.82 ± 159.94^d^

Quantitative variables were reported as mean ± SD

Dissimilar letters in each row indicate a significant difference of *P* < 0.05 between groups based on the ANOVA test and Duncan's multiple range tests (DMRT)

### Comparing macronutrients and amino acids intake of the participants based on BMI

In Table 4, macronutrients and amino acids intakes of the participants were compared based on BMI. Energy intake was different between lean and overweight, lean and obese, and normal and obese participants. The carbohydrate intake was different between lean participants and normal participants, different between normal and obese and lean participants, and between obese and normal and lean participants. In addition, protein intake showed a difference between lean, overweight and obese participants, on the one hand, and normal and obese participants, on the other hand. It could be observed that fat intake is different between obese and lean, normal and overweight participants. Intake of amino acids was significant among the groups. This way, tryptophan and phenylalanine intake was significantly different between lean and obese participants, and between overweight and obese participants. The intake of threonine, cystine and tyrosine was significantly different between obese and lean, and between normal and overweight participants. The intake of leucine, isoleucine, and lysine was significantly different between lean and overweight and obese participants, between normal and obese participants and between overweight and obese participants. The intake of valine and histidine was significantly different between lean and overweight, between lean and obese participants, and also between normal and obese participants.

**Table 4:** Comparing macronutrients and amino acids intake of the participants based on BMI

Variables	BMI			
	Lean (*N* = 12) (BMI < 18.5)	Normal (*N* = 111) (BMI = 18.5- 24.9)	Overweight (*N* = 38) (BMI = 25–29.9)	Obese (*N* = 11) (BMI ≥ 30)
*Macronutrients*				
Energy (kcal/day)	1758.73 ± 320.86^a^	2120.93 ± 612.80^ba^	2293.74 ± 558.52^cb^	2800/15 ± 737/70^d^
Carbohydrate (g/day)	256/03 ± 55/90^a^	313/34 ± 91/11^b^	337/05 ± 84/92^cb^	380/80 ± 73/79^dc^
Protein (g/day)	62/67 ± 15/08^a^	79/70 ± 26/84^ba^	87/88 ± 25/67^cb^	107/43 ± 42/33^d^
Fat (g/day)	58/23 ± 12/69^a^	68/38 ± 27/37^a^	73/30 ± 22/81^a^	100/51 ± 39/40^b^
*Amino acids intakes*				
Tryptophan (mg/day)	576/39 ± 279/68^a^	803/28 ± 396/28^ba^	921/84 ± 410/40^cb^	1072/71 ± 514/17^db^
Phenylalanine (mg/day)	2265/98 ± 904/66^a^	2626/52 ± 1198/62^ba^	3350/92 ± 1428/63^cb^	2952/51 ± 1075/05^db^
Threonine (mg/day)	2151/44 ± 675/83^a^	2794/45 ± 1465/94^a^	3192/54 ± 1332/16^a^	5309/29 ± 5127/69^b^
Cystine (mg/day)	856/23 ± 339/99^a^	1053/53 ± 665/97^a^	1149/47 ± 511/85^a^	3506/05 ± 5585/46^b^
Tyrosine (mg/day)	1733/03 ± 727/96^a^	2743/54 ± 2118/68^a^	2941/47 ± 1632/70^a^	4906/69 ± 5120/80^b^
Leucine (mg/day)	3853/11 ± 1505/81^a^	5278/01 ± 2812/52^ba^	6045/04 ± 2322/12^cb^	8020/09 ± 5141/50^d^
Isoleucine (mg/day)	2364/63 ± 1097/83^a^	3315/02 ± 1712/23^ba^	3792/91 ± 1512/35^cb^	5087/73 ± 3424/14^d^
Lysine (mg/day)	3331/03 ± 1398/59^a^	4038/68 ± 1968/38^ba^	5002/98 ± 2070/04^cb^	6377/96 ± 2682/29^d^
Valine (mg/day)	2792/36 ± 1001/62^a^	3887/50 ± 2399/52^ba^	4382/19 ± 1731/20^cb^	5757/31 ± 3866/29^dc^
Histidine (mg/day)	1445/06 ± 426/87^a^	1792/37 ± 878/83^ba^	2077/83 ± 839/60^cb^	2562/65 ± 1164/33^dc^

Quantitative variables were reported as mean ± SD

Dissimilar letters in each row indicate a significant difference of *P* < 0.05 between groups based on the ANOVA test and DMRT

### The relationship between the intake of branched-chain and aromatic amino acids with the variables based on gender

As the results of Pearson's correlation in Table 5, no relationship between BCAA and aromatic amino acids and the variables, including sleep quality, sleep duration, physical and mental fatigue scales, and physical activity based on gender, was observed.

**Table 5:** The relationship between the intake of branched-chain and aromatic amino acids with the variables based on gender

Variables	Males (*N* = 43)		Female (*N* = 129)		Total (*N* = 172)	
	CC	*P* value	CC	*P* value	CC	*P* value
*Branched-chain amino acids* (mg/day)						
Sleep quality	− 0.012	0.941	− 0.025	0.778	0.009	0.911
Sleep duration (h)	− 0.016	0.921	0.043	0.627	0.020	0.794
Physical activity (MET-min/wk)	− 0.116	0.457	− 0.112	0.206	− 0.130	0.089
*Aromatic amino acids* (mg/day)						
Sleep quality	− 0.009	0.955	− 0.077	0.383	− 0.022	0.776
Sleep duration (h)	0.031	0.843	0.053	0.547	0.040	0.600
Physical activity (MET-min/wk)	− 0.119	0.449	− 0.108	0.222	− 0.130	0.089

*P* values resulted from the analysis of Pearson correlation

CC: correlation coefficient

Correlation is significant at the 0.05 level (2-tailed)

*P* value < 0.05 was considered significant

### The relationship between the intake of branched-chain and aromatic amino acids with the variables based on age

As the results of Pearson's correlation in Table 6, no relationship between BCAAs and aromatic amino acids and the variables, including sleep quality, sleep duration, physical and mental fatigue scales, and physical activity based on age, was observed.

**Table 6:** The relationship between the intake of branched-chain and aromatic amino acids with the variables based on age

Variables	Under 30 years old (*N* = 118)		Over 30 years old (*N* = 54)		Total (*N* = 172)	
	CC	*P* value	CC	*P* value	CC	*P* value
*Branched-chain amino acids* (mg/day)						
Sleep quality	− 0.021	0.825	0.157	0.257	0.009	0.911
Sleep duration (h)	0.002	0.984	0.072	0.607	0.020	0.794
Physical activity (MET-minutes/wk)	− 0.147	0.112	− 0.015	0.915	− 0.130	0.089
*Aromatic amino acids* (mg/day)						
Sleep quality	− 0.028	0.762	0.057	0.682	− 0.022	0.776
Sleep duration (h)	0.011	0.907	0.136	0.328	0.040	0.600
Physical activity (MET-min/wk)	− 0.138	0.136	− 0.026	0.854	− 0.130	0.089

*P* values resulted from the analysis of Pearson correlation

CC: correlation coefficient

Correlation is significant at the 0.05 level (2-tailed)

*P* value < 0.05 was considered significant

### The relationship between the intake of branched-chain amino acids and the variables based on BMI

As can be seen in Table 7, there was a positive, significant relationship between sleep duration and intake of BCAAs in those with normal BMI (*P* = 0.031, CC = 0.205). The same relationship was observed between physical activity and intake of BCAAs in obese participants (*P* = 0.012, CC = 0.721).

**Table 7:** The relationship between the intake of branched-chain amino acids and the variables based on BMI

Variables	Lean (*N* = 12) (BMI < 18.5)		Normal (*N* = 111) (BMI = 18.5–24.9)		Overweight (*N* = 38) (BMI = 25–29.9)		Obese (*N* = 11) (BMI ≥ 30)		Total (*N* = 172)	
	CC	*P* value	CC	*P* value	CC	*P* value	CC	*P* value	CC	*P* value
Sleep quality	0.314	0.320	0.074	0.438	0.004	0.981	− 0.289	0.389	0.009	0.911
Sleep duration (h)	0.315	0.319	0.205	**0.031**	0.124	0.459	− 0.437	0.179	0.020	0.794
Physical activity (MET-min/wk)	− 0.174	0.589	− 0.024	0.804	− 0.017	0.921	0.721*	**0.012**	− 0.130	0.089

*P* values resulted from the analysis of Pearson correlation

CC: correlation coefficient

* Correlation is significant at the 0.01 level (2-tailed)

*P* value < 0.05 was considered significant﻿. Bold values are significant

### The relationship between the intake of aromatic amino acids and the variables based on BMI

In Table 8, a positive significant relationship was observed between sleep duration and intake of aromatic amino acids in those with normal BMI (*P* = 0.02, CC = 0.22). In addition, a positive significant relationship was observed between physical activity and intake of aromatic amino acids in the obese participants (*P* = 0.009, CC = 0.74).

**Table 8:** The relationship between intake of aromatic amino acids and the variables based on BMI

Variable	Lean (*N* = 12) (BMI < 18.5)		Normal (*N* = 111) (BMI = 18.5–24.9)		Overweight (*N* = 38) (BMI = 25–29.9)		Obese (*N* = 11) (BMI ≥ 30)		Total (*N* = 172)	
	CC	*P* value	CC	*P* value	CC	*P* value	CC	*P* value	CC	*P* value
Sleep quality	− 0.296	0.351	0.052	0.587	0.009	0.957	− 0.265	0.431	0.022	0.776
Sleep duration (h)	0.305	0.335	0.220	**0.020**	0.128	0.443	− 0.474	0.141	0.040	0.600
Physical activity (MET-min/wk)	− 0.174	0.584	− 0.007	0.943	− 0.016	0.924	0.740	**0.009**	− 0.130	0.089

*P* values resulted from the analysis of Pearson correlation

CC: correlation coefficient

Correlation is significant at the 0.05 level (2-tailed)

*P* value < 0.05 was considered significant﻿. Bold values are significant

## Discussion

This study examined the relationship between the intake of BCAAs and aromatic amino acids and individuals' sleep quality based on BMI, gender, and age. The intake of BCAAs and aromatic amino acids positively correlated with the sleep duration of those with a normal BMI. These results are in line with our hypothesis that amino acids intake is probably associated with sleep quality components based on BMI.

The findings of this study showed that the intake of BCAAs and aromatic amino acids had a positive significant relationship with sleep duration, a component of the sleep quality scale, in the participants with a normal BMI. Also, carbohydrate intake was different between normal and obese and lean participants. In addition, protein intake was different between normal and obese participants. Previous studies showed significant relationships between sleep quality and the intake of carbohydrates [33] and protein [36]. Studies show that carbohydrate intake, carbohydrate type, and blood glucose index can affect sleep quality [37]. Contradictory data have been obtained on protein intake, most of which have studied the effect of tryptophan on sleep than the total protein intake. Some studies have shown that protein intake is low among insomniacs or short-sleepers due to the decrease in the body's access to the amino acid tryptophan [38]. Dietary carbohydrates improve sleep quality by releasing serotonin, which directly affects sleep [37]. Insulin leads to selective absorption of LNAA by muscles, increasing the ratio of tryptophan to LNAA. Since Tryptophan competes with LNAA for transfer to the brain [39], this change in the ratio may increase tryptophan in the brain. Serotonin levels in the brain can increase after consuming carbs [40]. These studies are in line with our results. As we found that carbohydrate intake was different between normal and obese and lean participants, also protein intake was different between normal and obese participants. Previous studies highlighted that aromatic amino acid (tryptophan) improves sleep quality. However, there is no mechanism to justify the relationship between BCAAs and sleep duration/quality [41].

The association between the intake of aromatic amino acids and BCAAs with the sleep duration/ quality in obese participants was negative and non-significant. The reason for the negative relationship between high BMI and sleeps quality is that high BMI has a negative effect on sleep which has been confirmed in other studies described below. A study in China on 3225 subjects aged 18 to 65 found that obesity negatively affects sleep quality in men [42]. Racial and cultural differences or differences in the number of samples can be the reason for the difference in the relationship between BMI and sleep quality based on gender [42]. Yeh and his colleagues have reported that binge eating partly mediates the relationship between worse sleep quality to higher BMI [43]. Another possible explanation is that a high-protein diet may add to plasma concentrations LNAAs, including valine, tyrosine, isoleucine, leucine, and phenylalanine [44].

The association between sleep duration and the intake of BCAAs and aromatic amino acids in both under and over 30 years old was positive and non-significant. However, the sleep quality in people under 30 years old was negative (non-significant). Since in this study, the majority of the participants under the age of 30 were women, and the fatigue scale was upper among them, it is possible that the relationship between these amino acids and sleep quality, based on gender, has been affected by fatigue. Studies have shown the difference in sleep quality in middle-aged people (ages 50–93 years) [45]. We observed no significant difference between the sleep duration/quality because, in this age range (29.81 ± 9.82), aging and related changes do not affect sleep duration/quality [45].

This study has several strengths. The validity and reliability of the FFQ questionnaire have been well established [46]. The fatigue scale was considered. Also, the relationship between the intake of branched-chain and aromatic amino acids and individuals' sleep quality is based on several variables including BMI, gender, and age. However, this study also has several limitations. Firstly, the study design was cross-sectional. As a result, causality cannot be conferred due to the observational nature of this study. Secondly, the effect of the ratio of tryptophan to LNAA amino acids on the level of tryptophan absorption from the blood–brain barrier, the type of consumed carbohydrate, and the glycemic index associated with it, and its effect on the amount of tryptophan absorption from the blood–brain barrier was not measured and evaluated. The ratio of tryptophan to LNAA and the glycemic index between groups with adequate sleep and inadequate sleep should be considered in future studies so that more accurate results can be obtained. Lastly, dietary data were collected using an FFQ questionnaire that is dependent on the subjects' memory, which might result in bias. Future studies with prospective designs are needed.

## Conclusion

In summary, a positive and significant relationship was observed between the intake of BCAAs and aromatic amino acids with sleep duration only in those participants with a normal BMI. It demonstrated that in individuals with normal BMI, amino acids, protein, and carbohydrate intake may affect sleep duration and with modification of these factors sleep quality may get better. More study is needed to confirm these findings.

## Data Availability

The data that confirm the findings of this study are available from Ariyo Movahedi, but restrictions apply to the availability of these data, which were used under license for the current study, so they are not publicly available. Data are available from the authors upon reasonable request and with permission from Ariyo Movahedi.
